# A Boveri perspective on cancer biomarker testing using artificial intelligence

**DOI:** 10.1038/s41698-026-01692-8

**Published:** 2026-09-08

**Authors:** Esther Conde, Susana Hernandez, Marta Alonso, Daniel Curto, Fernando Lopez-Rios

**Affiliations:** 1 Department of Pathology, Hospital Universitario 12 de Octubre, Universidad Complutense de Madrid, Molecular and Computational Pathology Group, Research Institute Hospital 12 de Octubre (imas12), CIBERONC, Madrid, Spain; 2 Department of Pathology, Hospital Universitario 12 de Octubre. Molecular and Computational Pathology Group, Research Institute Hospital 12 de Octubre (imas12), Madrid, Spain

**Keywords:** Biomarkers, Cancer, Computational biology and bioinformatics

## Abstract

Artificial intelligence (AI) can predict genomic alterations from histology, yet its adoption is slowed by a lack of trust. We argue that *deliberate morphology* (i.e., a cognitive understanding of histological features supported by standardized annotations) creates a bidirectional feedback loop between clinical practice and model outputs.We translate these observations into an actionable hypothesis for clinical and computational teams: that by enhancing explainability, deliberate morphology could facilitate the responsible deployment of AI biomarkers in oncology.

## Introduction

A lifelong friend of one of us (F. L-R), who trained in Boston with Dr Castleman in the 1960s, enjoys recounting how the renowned pathologist would entertain his clinical colleagues by correctly inferring a patient’s biochemical parameters solely from haematoxylin and eosin-stained slides. The secret behind this skill was revealed only at the end of the elective rotation, to the delight of the audience. As embellished as this anecdote may seem, it underscores the often-overlooked potential of phenotypic-genomic correlations. Those of us who trained before the advent of immunohistochemistry (IHC) remain concerned about the increasing cognitive offloading among pathologists, some of whom now rely almost exclusively on extensive IHC antibody panels or next-generation sequencing (NGS) assays to achieve highly precise tumour classification. As a result, the time invested in scrutinizing subtle morphological clues on slides may appear unnecessary. A similar pattern of disengagement is now emerging in clinical practice with the implementation of artificial intelligence (AI) algorithms^1,2^. On the one hand, professionals working in precision oncology often regard AI with scepticism and maintain their distance; on the other, AI developers tend to embrace their black boxes and deprioritize explainability^1^. Rebuilding trust between these communities is, for now, the most effective way to overcome this clinical practice gap^2,3^. In this Commentary, we outline several historical and contemporary examples to illustrate how morphology could facilitate the adoption and implementation of AI for cancer biomarker testing, as prediction of treatment response is regarded as one of AI’s most realistic future applications^4,5^. We then consolidate these observations into an actionable hypothesis intended to guide pathologists and AI developers in linking morphology to genomic alterations.

## Tales of human intelligence

In 1914, Theodor Boveri, the prominent German cytologist, in his book *Concerning the Origin of Malignant Tumours*, anticipated the importance of oncogenes and tumour-suppressor mechanisms (“the excessive proliferation could be explained by an excess of specific chromosomes,” “are then attributable to the concomitant loss of other chromosomes”) and their influence on tumour morphology, both in the cellular and stromal compartments (“the variable distribution of chromosomes […] that occur in cancer cells give rise to cells with an extreme range of nuclear sizes,” “one outcome of the altered metabolism is doubtless the frequent production of markedly different effects on the healthy surrounding tissue”)^6^. Accordingly, pathologists have sought to establish phenotypic-genomic associations, at times even foregoing the genetic component of this binomial or successfully transferring phenotypic patterns across contexts. The former is best exemplified by the discovery of *EGFR* mutations as predictive biomarkers of response to tyrosine kinase inhibition in lung adenocarcinomas. In a long-forgotten article published before the seminal reports of this discovery^7–9^, investigators noted that responders were more likely to have lung adenocarcinomas with a distinctive growth pattern (referred to as “bronchioloalveolar” at the time, and now classified as “lepidic”)^10^. Over the past two decades, numerous characteristic morphological features have been linked to actionable genomic alterations across tumours arising in diverse anatomical sites. Examples include psammoma bodies or signet-ring cells with *ALK*, *ROS1*, or *RET* fusions^11^; fusiform cells with *MET* mutations^11^; koilocytoid nuclei with *FGFR3* mutations^12^; extracellular mucin with *KRAS* or *BRAF* mutations^11,13^; papillary or micropapillary growth patterns and enlarged nuclei with *BRAF* mutations^11,14,15^; psammomatous calcifications with *IDH1* mutations^16^; immune desert stroma with large deletions on chromosome 9^17^; hepatoid cytoplasm with *STK11* mutations^18^; or variability in nuclear size (i.e., pleomorphism) and tumour-infiltrating lymphocytes with several forms of genomic instability, including chromothripsis^13,19–22^. The latter is perhaps the most *Boverian* of these observations, as the nuclear envelope must adapt to the catastrophic fragmentation of chromosomes. Widespread access to NGS and the suboptimal sensitivity and specificity of these associations have moderated the initial enthusiasm. Therefore, detailed histopathological annotation is typically available only in the initial reports but is largely lacking in subsequent clinical trials or academic series investigating the biomarker in question. Finally, two examples illustrate the cross-context manifestation of the same genomic alteration, irrespective of the tissue of origin. The first involves *NTRK* fusions and secretory morphology in tumours of the breast and the head and neck region. Secretory carcinomas of the breast are defined by *NTRK* fusions, whereas their salivary gland counterparts frequently contain this actionable alteration, although it is not as pathognomonic^23^. The second example is even more striking. A rare sarcoma defined by the *ASPSCR1::TFE3* fusion (alveolar soft part sarcoma) and a paediatric renal carcinoma (*TFE3*-rearranged renal cell carcinoma) can harbour the same rearrangement and share overlapping morphological features^24^.

## Morphology-informed AI

**Fig. 1:**
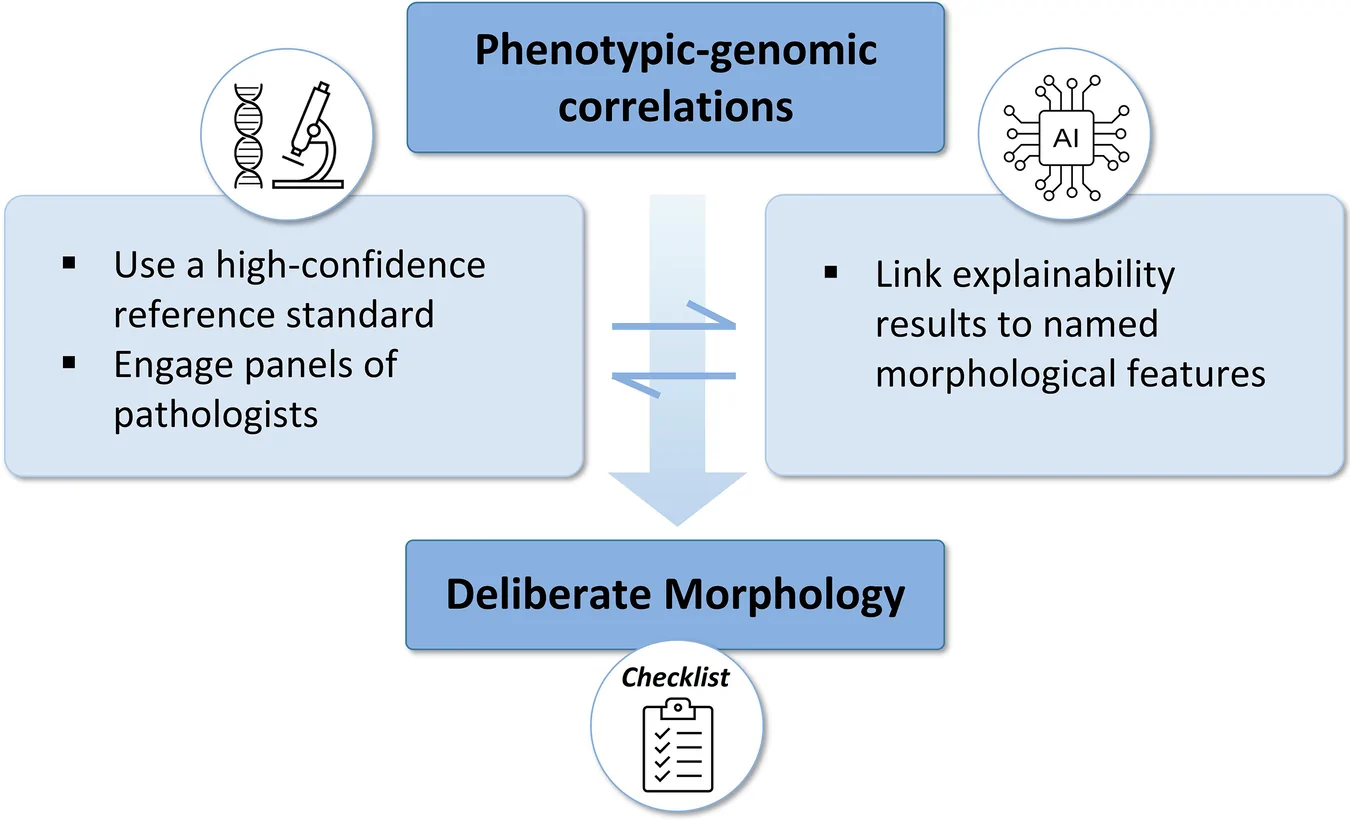
Phenotypic–genomic correlations and the relationship between clinical practice and AI. Deliberate morphology (i.e., a cognitive understanding of histological features supported by standardized annotations) provides opportunities for mutual benefit. On the clinical-practice side (left), reliable phenotypic–genomic correlations depend on using a high-confidence reference standard and on engaging panels of pathologists to capture the tacit morphological knowledge that underlies diagnosis. On the computational side (right), trust is built by linking model explainability outputs to named morphological features. The two perspectives are feed back on each other and converge on a checklist-based assessment of cellular and tissue-level features, which operationalizes the concept.

The Invisible Gorilla experiment is a classic demonstration of inattentional blindness, illustrating the failure to perceive an unexpected yet important stimulus when attention is focused elsewhere. In the original study, participants instructed to count basketball passes often failed to notice a person in a gorilla suit walking through the scene^25^. We invoke it here not to imply that an AI model can be distracted as a human observer might be, but as a metaphor: the morphological features we choose to annotate are constrained by the current classifications of human tumours^26^. In line with this notion, there is mounting evidence from many groups that most genomic AI predictors rely heavily on previously known distinctive phenotypes^27–38^. While the current approach is easy to reconcile with clinical practice, it is being challenged by the increasing opacity of foundation models^26,39,40^. How can we build trust in AI algorithms without a trade-off between accuracy and transparency, given that patients would choose the latter?^41^ Although common interpretability methods use maps to identify the most informative image tiles that drive the model’s final prediction, annotation practices often lack reproducibility and seldom provide comprehensive labelling across multiple spatial scales^42–44^. Our proposal is to dissect morphology and identify its cognitive basis (i.e., “we are least aware of what our minds do best”)^45–47^, by using a checklist to quantify all cellular features (nuclear and cytoplasmic size and shape, nuclear-to-cytoplasmic ratio, staining texture, membrane irregularity, presence and type of nuclear inclusions, nucleoli, mitotic figures, variability, and spatial arrangement) and tissue-level features (cellularity and stromal density, vascularity, immune infiltrate, fibrosis, tumour growth pattern, tumour–stroma interface, necrosis, and spatial heterogeneity), including IHC or immunofluorescence markers. By making explicit the tacit features that pathologists rely on, and by mapping AI explainability outputs onto these named morphological features, both human and machine reasoning can be audited and aligned^48,49^. This alignment through deliberate morphology offers two practical advantages: it helps verify that a model relies on biologically plausible features, and it supports the generation of reproducible datasets in which candidate associations can be tested prospectively (Fig. 1).

## Conclusions

A deliberate understanding of the morphological features driving histopathological diagnosis and the extreme prediction values produced by AI could facilitate the deployment of AI models for the identification of actionable biomarkers in patients with solid tumours, ultimately bridging computational efforts with biological plausibility. As we prepare to welcome AI as a new member of molecular tumour boards^50–52^, we all face a choice: to be part of the problem or to lead the responsible integration of AI into our biomarker testing workflows^53,54^.

## Data Availability

No datasets were generated or analysed during the current study.
